# The effect of carbohydrate sources: Sucrose, invert sugar and components of mānuka honey, on core bacteria in the digestive tract of adult honey bees (*Apis mellifera*)

**DOI:** 10.1371/journal.pone.0225845

**Published:** 2019-12-04

**Authors:** Michelle A. Taylor, Alastair W. Robertson, Patrick J. Biggs, Kate K. Richards, Daniel F. Jones, Shanthi G. Parkar

**Affiliations:** 1 Bee Biology & Productivity, Productive Biodiversity and Pollination, The New Zealand Institute for Plant and Food Research Limited, Hamilton, New Zealand; 2 Wildlife & Ecology, School of Agriculture and Environment, Massey University, Palmerston North, New Zealand; 3 Molecular Epidemiology & Public Health Laboratory, Infectious Disease Research Centre, School of Veterinary Science, Massey University, Palmerston North, New Zealand; 4 Bioinformatics and Statistics Group, School of Fundamental Sciences, Massey University, Palmerston North, New Zealand; 5 Statistical Science, Data Science, The New Zealand Institute for Plant and Food Research Limited, Auckland, New Zealand; 6 Bioinformatics, Molecular & Digital Breeding, The New Zealand Institute for Plant and Food Research Limited, Auckland, New Zealand; 7 Microbiome & Metabolism, Food Nutrition & Health, The New Zealand Institute for Plant and Food Research Limited, Palmerston North, New Zealand; Universitat Leipzig, GERMANY

## Abstract

Bacteria within the digestive tract of adult honey bees are likely to play a key role in the digestion of sugar-rich foods. However, the influence of diet on honey bee gut bacteria is not well understood. During periods of low floral abundance, beekeepers often supplement the natural sources of carbohydrate that honey bees collect, such as nectar, with various forms of carbohydrates such as sucrose (a disaccharide) and invert sugar (a mixture of the monosaccharides glucose and fructose). We compared the effect of these sugar supplements on the relative abundance of bacteria in the gut of bees by feeding bees from a single colony, two natural diets: mānuka honey, a monofloral honey with known antibacterial properties, and a hive diet; and artificial diets of invert sugar, sucrose solution, and sucrose solutions containing synthesised compounds associated with the antibacterial properties of mānuka honey. 16S ribosomal RNA (rRNA)-based sequencing showed that dietary regimes containing mānuka honey, sucrose and invert sugar did not alter the relative abundance of dominant core bacteria after 6 days of being fed these diets. However, sucrose-rich diets increased the relative abundances of three sub-dominant core bacteria, Rhizobiaceae, Acetobacteraceae, and *Lactobacillus kunkeei*, and decreased the relative abundance of *Frischella perrara*, all which significantly altered the bacterial composition. Acetogenic bacteria from the Rhizobiaceae and Acetobacteraceae families increased two- to five-fold when bees were fed sucrose. These results suggest that sucrose fuels the proliferation of specific low abundance primary sucrose-feeders, which metabolise sugars into monosaccharides, and then to acetate.

## Introduction

European honey bees (*Apis mellifera* L.) are the primary pollinators of numerous nut, fruit, and vegetable crops, so they play an integral part in global food production [1–4]. Pollination by honey bee species (*Apis* sp.) and other bee species also ensures reproductive success of uncultivated plants, including those in their native ranges [2, 4, 5]. In addition to pollination, honey bees also produce economically valuable honey, as well as acting as a source of bee products such as pollen and propolis, the waxy resin collected from leaf buds. All three products are utilised both as food and by the medicinal and dietary-supplement industries. This global utilisation of honey bees has made it important to understand the factors that influence honey bee health. Hive management practices, and the colony’s access to adequate nutritional resources, is crucial to colony health. The health and production of a colony is dependent on the location that beekeepers place their hives to forage, the supplementary carbohydrate and protein sources they feed their bees, and when they do this [6–8].

Honey bees require carbohydrate sources that they naturally obtain from nectar. Nectar predominantly consists of water, pollen, and varying proportions of the monosaccharides glucose and fructose, and the disaccharide sucrose [9–11]. Bee-pollinated flowers tend to produce nectar with >35% sugar and honey bees reduce the moisture content within nectar to about 17% (range 13–24%) resulting in honey with a concentrated mix of sugar comprising of about 69% monosaccharides (approximately 38% fructose and 31% glucose) [12], and <15% disaccharide (sucrose) [11].

The carbohydrates in the honey bee diet may be absorbed by the gut to sustain the bees, or metabolised by gut bacteria before absorption [13]. However, during winter and spring when nectar can be scarce, and when preparing colonies for winter, beekeepers often feed their bees supplementary carbohydrates. These include sucrose, invert sugar (a mix of glucose and fructose) and high fructose corn syrup (HFCS; a sweetener made from cornflour, in which some glucose has been converted to fructose) [6, 14, 15]. This additional feeding often protects the bees from malnutrition, which can lead to immune system impairment [16] and increased pesticide susceptibility [17]. However, extensive feeding of either sucrose or HFCS causes significant differences in gene expression by the honey bee fat body (the nutrient-sensing organ responsible for nutrient storage), including those associated with energy metabolism, and antimicrobial peptide production [18]. These epigenomic consequences in honey bees, are very similar to sugar-associated disrupted metabolism seen in vertebrates that are supplemented with either glucose or fructose [19].

The function of bacteria residing in the digestive tract of animals, honey bees included, is a rapidly developing field of scientific research that is proving to be fundamental to animal health [20, 21]. A meta-analysis of the composition of gut bacteria in 62 insect species suggest bacterial similarity within the subfamily Apinae, as well as the distinct communities of *A*. *mellifera* relative to other bees [21]. This meta-analysis suggests that bacterial community structure in insects may be influenced by diet [21]. However, as this was not specifically identified for honey bees, and recent research predominantly focusses on the effect of pollen rather than carbohydrates, and does not specify the type or amount of supplementary feed consumed [22–24], the effect of carbohydrate diets on the bacterial composition in the honey bee gut, and how this may influence bee health, has not yet been researched.

The microbiota within the gut of adult worker honey bees contain 8 to 10 core bacterial phylotypes [25]. These phylotypes are rarely found outside of the honey bee gut and are considered part of the conserved core microbiota, albeit with different relative abundances and being more or less frequently detected [23, 26]. The dominant core phylotypes consist of two species from the phylum Proteobacteria, *Gilliamella apicola* and *Snodgrassella alvi* [27]; two clusters of species from the phylum Firmicutes, *Lactobacillus* Firm-4; *Lactobacillus* Firm-5 [28, 29]; and the species cluster in the phylum Actinobacteria, *Bifidobacterium* [30]. The relative abundances of the remaining core phylotypes are less consistent, and not always detected: *Frischella perrara* [31], *Bartonella apis* [32], *Parasaccharibacter apium* [33], and a Gluconobacter-related species group designated Alpha2.1 [29].

The gut has several sections that each contain bacterial populations of different taxonomic compositions [28]. Only a few bacteria reside in the crop and the midgut. These include core species that also reside in the larval gut such as Rhizobiaceae, the nitrogen-fixing bacteria [34], Acetobacteraceae and *Lactobacillus kunkeei* [35]. The adult ileum is dominated by the non-sugar fermenter *S*. *alvi* that colonises the gut wall, and the sugar fermenter *G*. *apicola* that resides in the lumen [36]. The distal rectum is dominated by *Lactobacillus* and *Bifidobacterium* [37, 38].

Bacteria in the honey bee gut are often symbiotic residents, with functions likely to be essential to bee nutrition, digestion, reproduction, and protection against toxins and pathogens [39–41]. Metatranscriptome sequencing has shown that bacteria play several critical roles in metabolising carbohydrate substrates. Some of these bacteria are primary sucrose-feeders, and metabolise sugars into monosaccharides that are further metabolised into acid metabolites such as acetate and lactate that assist with the breakdown of toxic sugars [41, 42]. The gut bacteria thus contribute to the repertoire of enzymes required for carbohydrate digestion [43]. The bacterial species from the phyla Actinobacteria and the class Bacilli produce several glycoside hydrolases, which in turn break down complex polysaccharides and simple sugars, and also produce peptidases for protein hydrolysis [41]. In particular, glycoside hydrolase family 32 was found to be linked with sucrose degradation [41].

Sucrose solutions and honey are both antibacterial *in vitro* because of osmolytic effects when applied at concentrations ≥40% and 10–20% (v/v), respectively [44, 45]. The anti-bacterial properties of honey have been attributed to this high sucrose-equivalent concentration ca. 80% (v/v), as well as the presence of hydrogen peroxide, produced by the enzyme glucose oxidase that the bees add to nectar [44]. Mānuka honey, obtained from the plant *Leptospermum scoparium*, comprises ca. 85% sugars, predominantly fructose and glucose, with < 1–15% sucrose [46–48]. Mānuka honey demonstrates peroxide activity, but methylglyoxal (MGO) is the primary antibacterial compound at concentrations >0.15 mg/g [45, 49–51]. This was characterised by comparing the bactericidal effects of honey containing high MGO with the effects of sucrose on resistant strains of Gram-negative Gammaproteobacteria (*Escherichia coli* and *Pseudomonas aeruginosa*) and Gram-positive organisms (*Bacillus subtilis*, *Staphylococcus aureus*, *Enterococcus faeciumas*) [45]. MGO is derived from the breakdown of dihydroxyacetone (DHA), which is also found in high concentrations in mānuka honey [50, 52, 53]. The concentration of MGO in mānuka honey less than one year old is normally between 0.10 and 0.79 mg/g. This can increase to 1.54 mg/g with the breakdown of DHA over the course of a year, or if the honey has been heat treated [51].

Honey bees commonly consume carbohydrates in the form of nectar, honey, sucrose, and invert sugar, but not all carbohydrates are utilised by bees or their microbial residents [18, 54]. We hypothesise that honeys will affect the diversity and relative abundance of bacteria present in the digestive tract compared with sucrose solutions, and that these effects may be attributed to the differences in the sugar composition in these diets. We used 16S rRNA gene sequencing to investigate the effect of carbohydrate sources on the relative abundance of bacteria present in the digestive tract of caged adult honey bees from a single colony. The effect of two different mānuka honeys (predominantly monosaccharides), were compared with the effect of invert sugar (mix of monosaccharides), sucrose (a disaccharide), and diets containing the mānuka associated chemicals MGO and DHA in sucrose solution. These were also compared with the effects of diet consumed by caged bees in a hive.

## Materials and methods

### Honey bee sampling and their treatment diets

A single *A*. *mellifera* colony, located at The New Zealand Institute for Plant and Food Research Limited (PFR), Hamilton, New Zealand (NZ), was used in this trial to limit the effect of genetic variation. A single frame of black-eyed (18–20 days old) honey bee pupae was selected from a colony in early summer (December 2017) and incubated at 33°C and 65% relative humidity (RH). Throughout a 70-h period, a total of 1050 newly emerged workers, which were <24 h old were marked on their abdomen with a spot of nail polish, caged and returned to the parent colony for at least seven days. This allowed colonisation of the digestive tract with a full complement of bacteria, as observed by Powell, Martinson [38]. The bees slowly released themselves from the cages over 24 h as the grass blocking the entrance dehydrated. Ten days after the first marked bees were returned to their colony, 7- to 10-day-old marked bees were recaptured from the colony and ten bees were placed in each plastic queen cage (75 x 30 x 15mm). It took approximately 4 h to set up the seven diet treatments so replicate cages were allocated to each of the treatments sequentially, one cage per treatment. The six modified diets had eight replicates and the hive control diet had five replicates. In total, there were 53 cages of bees (Table 1).

**Table 1:** Carbohydrate diets fed to honey bees.

Treatment code	Cage replicates	Diet	Sucrose (%)	MGO (mg/kg)	DHA (mg/kg)
H	5	Hive diet: honey frame above the brood nest	Unknown^ʘ^	_	_
IS	8	20 ml of 67°B bulk invert sugar (NSFGIVB5BULK)	0	_	_
S	8	20 ml of 50% sucrose solution	50	_	_
MH15	8	20 g of 100% mānuka honey from 2015	<1–15 ‡	745	1238
MH17	8	20 g of 100% mānuka honey from 2017	<1–15 ‡	394	1692
MGO	8	20 ml of 745 mg MGO/kg 50% sucrose solution*	~50	745	_
DHA	8	20 ml of 1692 mg DHA/kg 50% sucrose solution	~50	_	1692

^ʘ^ The hive was not fed supplementary sources of sucrose throughout the spring.

‡ Percent sucrose (w/w) was based on mānuka honey analysis in the literature [46–48].

* 0.931 ml 40% aqueous MGO + 499.17 ml 50% S).

Feeding commenced immediately and continued for a duration of 6 days. The control cages (H) were pressed into the wax and honey in a honey frame above the brood nest of the parent hive. The bees consumed the honey *ad libitum* and were likely to have received food from the hive bees. The remaining six treatments were fed to the bees *ad libitum*, through gravity feeders and the cages were incubated at 33°C and 65% RH for 6 days. These laboratory treatments were refreshed after 3 days. Two treatments were two mānuka honeys harvested by Hikutaia Honey (Opotiki, NZ) from the same apiary, but from different seasons: mānuka honey from the 2015 harvest (MH15, Lot # 112–15), and mānuka honey from the 2017 harvest (MH17, Lot # 49–17). These honeys were extracted from the wax frames at 33°C, and then the honeys were passed through a 1200 μm mesh. Prior to the trial, the honeys were analysed for DHA and MGO by Analytica Laboratories (Hamilton, NZ). Two further treatments were 50% (w/w) sucrose solution mixed with one of two chemically synthesised mānuka components: 1692 mg/kg DHA (Sigma D107204, Lot # MKBS8481V, Sigma-Aldrich, Auckland, NZ) or 745 mg/kg MGO (Sigma M0252, Lot # BCBK5800V, Sigma-Aldrich, Auckland, NZ). The concentrations tested were the maximum concentrations observed in the analysed mānuka honeys (MH15 and MH17) and previously reported in the literature [55]. Two more treatments were supplementary carbohydrate solutions used by the beekeeping industry: 67°B invert sugar (IS), and 50% sucrose solution (w/w) (S).

Sixteen days after their emergence as adults, 100% of the caged bees were still alive. At that point, five individuals from each of the 53 cages (a total of 265 bees) were placed in 90% ethanol and stored at –70°C.

### DNA extraction, amplification, and 16S rRNA gene sequencing

For each replicate the five stored bees were thawed for three minutes and then each digestive tract (crop to rectum) was aseptically dissected and pooled into a single DNase- and RNase-free ZR BashingBead^™^ Lysis Tube, in ice, containing 750 μl lysis solution. At this point, the tubes were returned to –70°C until processing as the lysis solution contained a proprietary DNA stabilising agent. The pooling was conducted to ensure homogeneity of the sample extracted, (given that an individual gut sample averaged 26.3 mg such a low biomass would have yielded a low concentration of DNA which may have been insufficient for sequencing), and enabled the inclusion of more biological replicates. The five pooled tracts were processed for DNA extraction using the Zymo Research Quick-DNA^™^ Fecal/soil Microbe Miniprep kit (Zymo Research Corporation (ZR), California, USA). The samples were homogenised at 6 m/s for 40 seconds using a FastPrep®-24 (MP Biomedicals, Seven Hills, Australia), and then the rest of the ZR protocol was followed. The eluted DNA samples were stored at –70°C prior to being sent on ice by overnight courier to the Massey Genome Service (MGS; Massey University, Palmerston North, NZ) for 16S rRNA gene sequencing of the V3V4 hypervariable region [56].

MGS evaluated the DNA concentration in each sample with Qubit^™^ 2.0 Fluorometer (ThermoFisher Scientific, NZ) analysis using a dsDNA HS Assay Kit for 12 samples per plate. A PCR reaction was then performed using primers with adaptors: 16Sf_V3 (5' - 3' direction)–CCTACGGGAGGCAGCAG; and 16Sf_V4 (5' - 3' direction)–GTGCCAGCMGCCGCGGTAA [56]. The PCR products (c. 420–440 base pairs) were purified to generate a library and their concentrations were analysed using Qubit^™^. The products were pooled in equimolar concentrations and the concentration and size were confirmed with both Qubit^™^ and LabChip (PerkinElmer, Waltham, MA, USA) analysis. The PCR products were sequenced with a 250-base paired end run on an Illumina MiSeq^™^ platform (Illumina Inc.) with version 2 chemistry. Illumina PhiX Control v3 (FC-110-3001) was included as the sequencing control. The resulting sequences are available in the National Center for Biotechnology Information’s (NCBI’s) Sequence Read Archive (PRJNA531038).

### Sequence processing and characterisation of microbial communities

A total of 5,127,987 read pairs were detected across all seven treatments and cage replicates. The Illumina de-multiplexed fastq sequence data were processed and trimmed using ea-utils to a 0.01 probability of error, an equivalent Phred score of Q20 [57], then further processed using the Quantitative Insights Into Microbial Ecology 2 (QIIME 2) analysis suite, version 2018.2 [58] (https://github.com/PlantandFoodResearch/bioinf_Apis_metabarcoding). The reads were run through dada2 methodology in QIIME2 to filter and trim the paired-end sequences, dereplicate them, and filter chimeras to produce exact amplicon sequence variants.

The honey bee microbiome is a relatively new area of research, with new bacterial strains being identified and reclassified frequently. Previous work indicated that some sequences were incorrectly assigned to old nomenclature. To ensure taxonomic classification of honey bee gut bacteria were current, the 16S rRNA BLAST (Basic Local Alignment Search Tool) database was downloaded from NCBI (ftp://ftp.ncbi.nlm.nih.gov/blast/db/) and customised to make a QIIME 2 compatible reference dataset (https://github.com/pjbiggs/16SrRNA_taxonomy). From the dataset a biological observation matrix (BIOM) was created that contained the Operational Taxonomic Units (OTUs) identified from the sequencing of each sample, that matched with the assigned taxonomy. Any OTUs that were unable to be identified taxonomically to species level were assigned to the closest identified taxonomic level.

### Statistical analyses

Phylogenetic diversity was measured within a sample (α-diversity), and between samples (β-diversity) using the web-based tool MicrobiomeAnalyst [59, 60]. The data counts were filtered to a minimum of two, as well as a 10% prevalence in the samples. Variance was filtered using a 10% coefficient of variation. To reduce estimation errors that result from the different number of sequences per sample, the data were rarefied to 52880, the number of sequences in the smallest sample. The data were scaled using total sum but were not transformed.

Alpha-diversity was calculated at the feature level using Kruskal-Wallis pairwise comparisons of four diversity measures: Observed OTUs, Chao1, Shannon, and Simpson. β-diversity for the taxonomic level feature was calculated using the distance methods Bray-Curtis dissimilarity (uses abundance of each OTU) and Jaccard Index (presence/absence), and the differences between the samples were compared using a permutational multivariate analysis of variance (PERMANOVA) [61]. 3-D plots of Principal Coordinates Analysis (PCoA) were used to present β-diversity.

Further data analysis was conducted in R (version 3.5.1) [62]. For all analyses, sequences with a minimum total read composition of <0.1% prevalence were filtered from the dataset (the remaining number of reads totalled 4,767,519). To investigate the differences in the number of phylotypes between treatments, a Poisson generalised linear model was used with the number of phylotypes as the response and treatment as a fixed effect. To explore the relationship between phylotypes and treatment, the data were visually explored using heat maps, where the response was the mean read composition per replicate. The interaction of the relative abundance (proportion of total bacterial abundance) of phylotypes was explored using nonmetric multidimensional scale (NMDS) plots. For the NMDS plots, the dissimilarity matrix was calculated using the Bray-Curtis dissimilarity method. A linear mixed effect regression model was performed using the R package lme4 [63]. Replication was included as a random effect to account for replicate to replicate variability between all phylotypes present within each sample. Assumptions were checked via standard residual plots and a logarithmic transformation was applied. Post-hoc pairwise comparisons of least-square means were carried out using Tukey. The predicted means were back-transformed and dissimilar letters were used to indicate significant differences among treatments. To determine whether carbohydrate diet altered the bacterial community within the gut, a mixed model PERMANOVA [61] was conducted using Adonis2 [64] to compare the variation in relative abundance between the treatments.

## Results

The 4,767,519 read pairs detected across all seven treatments and cage replicates were clustered into 75 OTUs. OTU sequences were classified as 11 unique phylotypes, of which two were families, one was a genus, and eight were species (Table 2). The mean number of OTUs listed in Table 2 were similar for each diet treatment but the invert sugar (IS) treatment had the least (69 OTUs). Further analysis of this difference in OTUs revealed no clear pattern, only that the IS treatment had one less OTU for each of five phylotypes (S1 Table) and the Poisson generalised linear model provided no evidence to suggest a difference in the number of phylotypes between treatments (S2 Table). Similarly, the α-diversity analysis indicated that none of the treatments caused a significant influence on the richness (Chao1, Observed OTUS), and this did not change after accounting for evenness (Shannon and Simpson Indices) (P > 0.05) (S2 Table).

**Table 2:** Number of Operational Taxonomic Units (OTUs) and the associated taxonomic groups within the gut of NZ honey bees.

Diet treatment	Number of OTUs	Number of phylotypes
H	74	11
S	75	11
IS	69	11
MH15	72	11
MH17	72	11
MGO	74	11
DHA	74	11

The bees were sourced from a single hive and fed different carbohydrate diets for 6 days: Hive-fed (H); sucrose (S); invert sugar (IS); 2015 mānuka honey (MH15); 2017 mānuka honey (MH17); methylglyoxal (MGO); dihydroxyacetate (DHA).

The phylotype *Lactobacillus* sp. dominated all the samples with counts 3- to 4-fold higher than all other phylotypes. The25 OTUs associated with *Lactobacillus* sp. suggests the phylotype contains a lot of genetic diversity (Table 3). In comparison, the three species that were identified as *Lactobacillus* species: *L*. *mellis*, *L*. *mellifer*, and *L*. *kunkeei*, contained 8, 1, and 1 OTUs, respectively. *Lactobacillus mellifer* is often included in the phylotype *Lactobacillus* Firm-4. However, this manuscript individually identifies *L*. *mellifer* and refers to *Lactobacillus* Firm-4 as the phylotype *Lactobacillus* sp.

**Table 3:** Mean relative abundance for each of the phylotypes in the digestive tract of honey bees fed different carbohydrate diets for 6 days.

Bacterial phylotype	Mean OTUs	H	IS	MH15	MH17	S	MGO	DHA
*Lactobacillus* sp.*	25	42.6^a^	51.5^a^	44.4^a^	46.8^a^	44.7	48.9	46.4
		^(25.8–70.4)^	^(34.7–76.5)^	^(29.9–65.9)^	^(31.5–69.6)^	^(30.1–66.4)^	^(32.9–72.7)^	^(31.2–68.9)^
Gilliamella apicola*	13	14.0^a^	11.7^a^	15.1^a^	17.7^a^	10.1^a^	10.2^a^	10.1^a^
		^(8.5–23.0)^	^(7.9–17.4)^	^(10.2–22.5)^	^(11.9–26.3)^	^(6.8–15.0)^	^(6.9–15.1)^	^(6.8–15.0)^
*Lactobacillus mellis*	11	11.9^a^	9.3^a^	8.7^a^	7.8^a^	10.5^a^	10.3^a^	9.5^a^
		^(7.2–19.7)^	^(6.3–13.9)^	^(5.8–12.9)^	^(5.2–11.6)^	^(7.0–15.5)^	^(6.9–15.3)^	^(6.4–14.1)^
*Bifidobacterium*	8	8.5^a^	8.0^a^	8.3^a^	7.7^a^	8.5^a^	6.8^a^	7.7^a^
coryneforme*		^(5.1–13.9)^	^(5.4–11.9)^	^(5.6–12.4)^	^(5.2–11.5)^	^(5.8–12.7)^	^(4.6–10.2)^	^(5.2–11.4)^
Snodgrassella alvi*	5	8.0^a^	6.3^a^	8.0^a^	6.2^a^	4.8^a^	5.9^a^	5.2^a^
		^(4.9–13.2)^	^(4.2–9.3)^	^(5.4–11.0)^	^(4.2–9.3)^	^(3.2–7.1)^	^(3.2–7.1)^	^(3.5–7.8)^
*Ensifer adhaerens*	1	1.4^a^	1.2^a^	2.0^a^	2.0^a^	2.4^a^	2.4^a^	2.1^a^
		^(0.8–2.2)^	^(0.8–1.7)^	^(1.4–3.0)^	^(1.3–2.9)^	^(1.6–3.5)^	^(1.6–3.5)^	^(1.4–3.1)^
Lactobacillus mellifer*	1	1.5^a^	1.3^a^	1.8^a^	1.5^a^	1.9^a^	1.9^a^	1.8^a^
		^(0.9–2.5)^	^(0.9–2.5)^	^(1.2–2.7)^	^(1.0–2.2)^	^(1.3–2.8)^	^(1.3–2.8)^	^(1.2–2.7)^
*Frischella perrara*	5	5.5^b^	2.7^ab^	4.1^ab^	3.6^ab^	**2.0**^**a**^	**2.7**^**ab**^	**2.0**^**a**^
		^(3.4–9.1)^	^(1.8–4.0)^	^(2.8–6.1)^	^(2.4–5.4)^	^(1.3–2.9)^	^(1.8–4.0)^	^(1.4–3.0)^
Rhizobiaceae	4	1.1^a^	0.8^a^	1.4^a^	0.8^a^	**4.3**^**b**^	**4.1**^**b**^	**5.0**^**b**^
		^(0.6–1.7)^	^(0.6–1.2)^	^(1.0–2.1)^	^(0.6–1.2)^	^(2.9–6.3)^	^(2.7–6.0)^	^(3.4–7.5)^
Acetobacteraceae	1	1.3^a^	1.5^a^	2.0^ab^	2.5^ab^	**4.1**^**b**^	3.2^ab^	3.3^ab^
		^(0.8–2.2)^	^(1.0–2.2)^	^(1.3–2.9)^	^(1.7–3.7)^	^(2.8–6.1)^	^(2.2–4.8)^	^(2.2–4.9)^
*Lactobacillus kunkeei*	1	0.3^ab^	0.3^a^	0.2^a^	0.1^a^	**0.8**^**b**^	0.2^a^	**0.7**^**b**^
		^(0.2–0.5)^	^(0.2–0.4)^	^(0.1–0.3)^	^(0.1–0.3)^	^(0.5–1.2)^	^(0.1–0.3)^	^(0.5–1.1)^

Honey bees from a single hive were fed one of seven carbohydrate diets for six days: Hive-fed (H), invert sugar (IS), 2015 mānuka (MH15), 2017 mānuka (MH17), sucrose (S), methylglyoxal (MGO), and dihydroxyacetate (DHA). The columns of sucrose-rich treatments are shaded in light grey. The back transformed means were identified using Tukey post-hoc comparisons from the linear mixed effect model, α = 0.05. The dissimilar letters indicate significant differences among treatment means. Differences are bolded and the phylotypes that changed significantly with diet are shaded in dark grey. The corresponding phylotypes are shaded in medium grey. OTUs (Operational Taxonomic Units). Dominant core bacteria (*).

**Fig 1:**
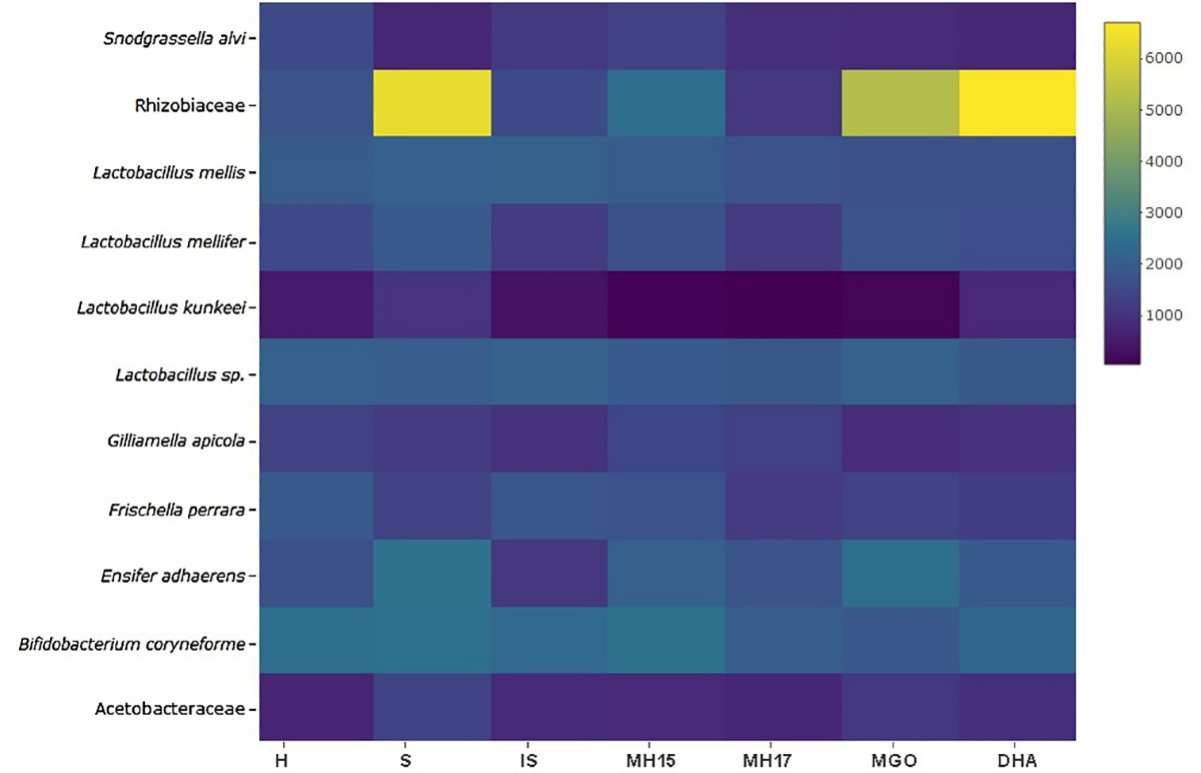
**Heatmap of mean composition reads of the bacteria in the honey bee digestive tract fed different carbohydrate diets.** Reads >0.1% prevalence were included. Honey bees from a single hive were fed one of the following treatments for 6 days: Hive-fed (H), sucrose (S), invert sugar (IS), 2015 mānuka (MH15), 2017 mānuka (MH17), methylglyoxal (MGO), and dihydroxyacetate (DHA).

Although each diet produced very similar gut microbiome diversity and most of the core bacteria were found at similar relative densities across all diets, there is evidence that the proportion of some phylotypes changed in response to diet (Table 3, Fig 1). The heatmap demonstrates evidence of sucrose treatments (S, MGO, and DHA) affecting the mean composition reads for some of the phylotypes, such as Rhizobiaceae (Fig 1). The effect of diet was supported by the Analysis of Deviance for the linear mixed effect regression model where significant interaction between the mean relative abundance of each bacteria within each treatment was evident (*P* < 0.001) (S2 Table).

The effect of the different carbohydrate diets on the phylotypes was further identified with the post-hoc pairwise comparisons where the mean relative abundance of four of the sub-dominant core phylotypes differed significantly (*P* < 0.01) (Table 3) (see S1 Table for the mean total abundance for each bacteria within each treatment. The totals in the S1 Table decreased in the same order as Table 3): The relative abundance of *Frischella perrara* was two-fold higher (*P* < 0.01) in the hive control than in the sucrose and DHA treatments. The relative abundance of Rhizobiaceae was 4- to 5-fold higher (*P* < 0.01) in the three sucrose-rich treatments (sucrose, MGO and DHA) than in the four sucrose-poor treatments (H, IS, MH15, MH17). Acetobacteraceae was also 2- to 3-fold higher (*P* < 0.01) in the sucrose treatment than the hive and invert sugar treatments, while the relative abundance of *L*. *kunkeei* was 2- to 7-fold higher (*P* < 0.01) in the sucrose-rich and DHA treatments compared with the MGO, invert sugar, and mānuka honey treatments. In contrast, the diet treatments did not affect the relative abundance of the five dominant core bacteria (*G*. *apicola*, *S*. *alvi*, *Lactobacillus* sp., *Lactobacillus* Firm-5, and *Bifidobacterium*).

**Fig 2:**
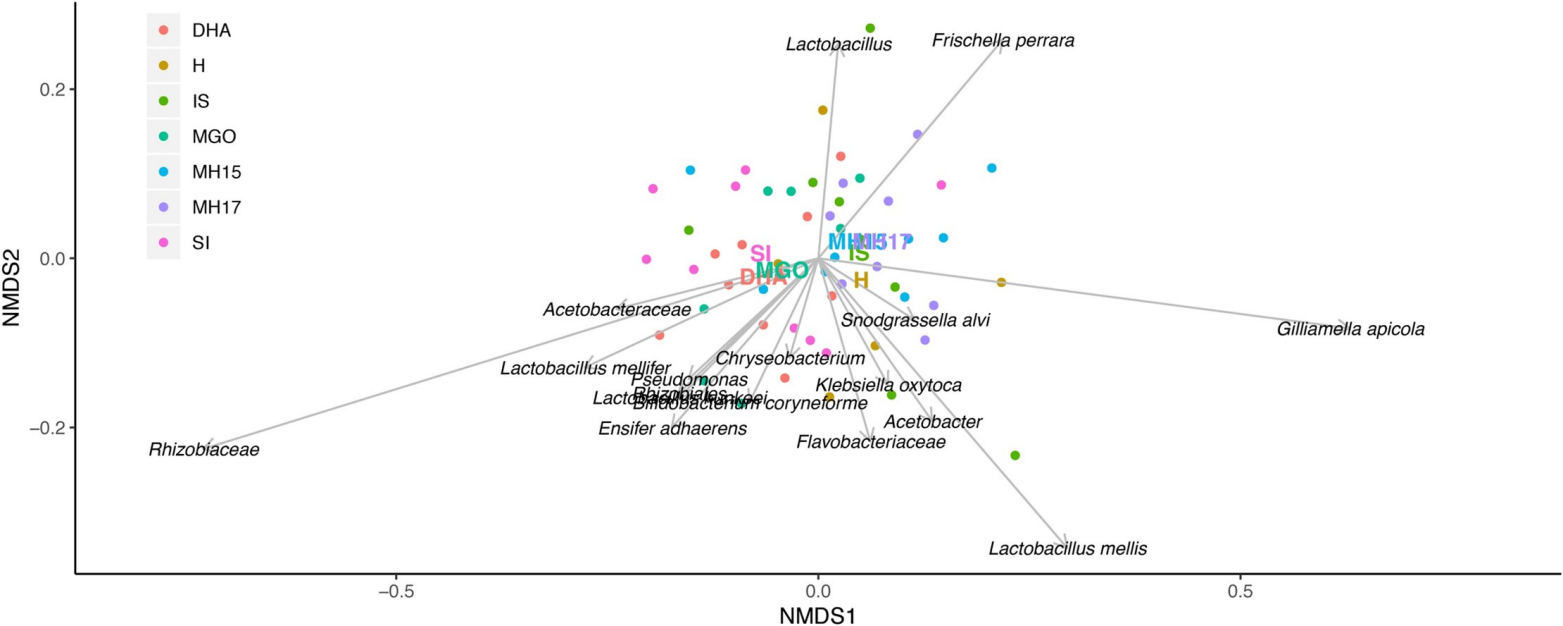
**Nonmetric multidimensional scaling plot of relative abundance of bacteria in the digestive tract of honey bees fed different carbohydrate diets.** Total read composition with >0.1% prevalence was included. Honey bees from a single hive were fed one of the following treatments for 6 days: Hive-fed (H), sucrose (S), invert sugar (IS), 2015 mānuka (MH15), 2017 mānuka (MH17), methylglyoxal (MGO), and dihydroxyacetate (DHA). A solution for the plot was reached at stress level 0.273.

The NMDS analysis (Fig 2) also suggests that the composition of the microbiome shifted primarily as a function of the sucrose content of the diet. Communities in the sucrose-rich diets (S, MGO, and DHA) were displaced from the sucrose-poor diets (H, IS, MH15, MH17) along axis one of the ordination. The sucrose-rich diets produced communities that converged with a strong representation of Rhizobiaceae, while the sucrose-poor diets tended to increase in *G apicola*. The relative abundance of *F*. *perrara* and *L*. *mellis* tended to move towards the opposite direction on axis two, and thus seemed to be less affected by sucrose content or other contents of the diet.

The PERMANOVA confirmed significant differences in community assembly with diet for both distance measures (*P* < 0.001, R^2^ = 0.243) (Table 4).

**Table 4:** The effect of dietary treatments on the beta-diversity of OTUs within the gut of NZ honey bees.

**Distance method**	**P—value**	**F- value**	**R squared**	**Axis 1**	**Axis 2**	**Axis 3**
Bray-Curtis	< 0.001	1.7153	0.1828	15.8%	8.7%	8.5%
Jaccard	< 0.001	1.4539	0.1594	11.3%	6.5%	6.3%

Honey bees from a single hive were fed one of seven treatments for 6 days. The relative abundance of OTUs were analysed with different distance methods using PERMANOVA.

**Fig 3:**
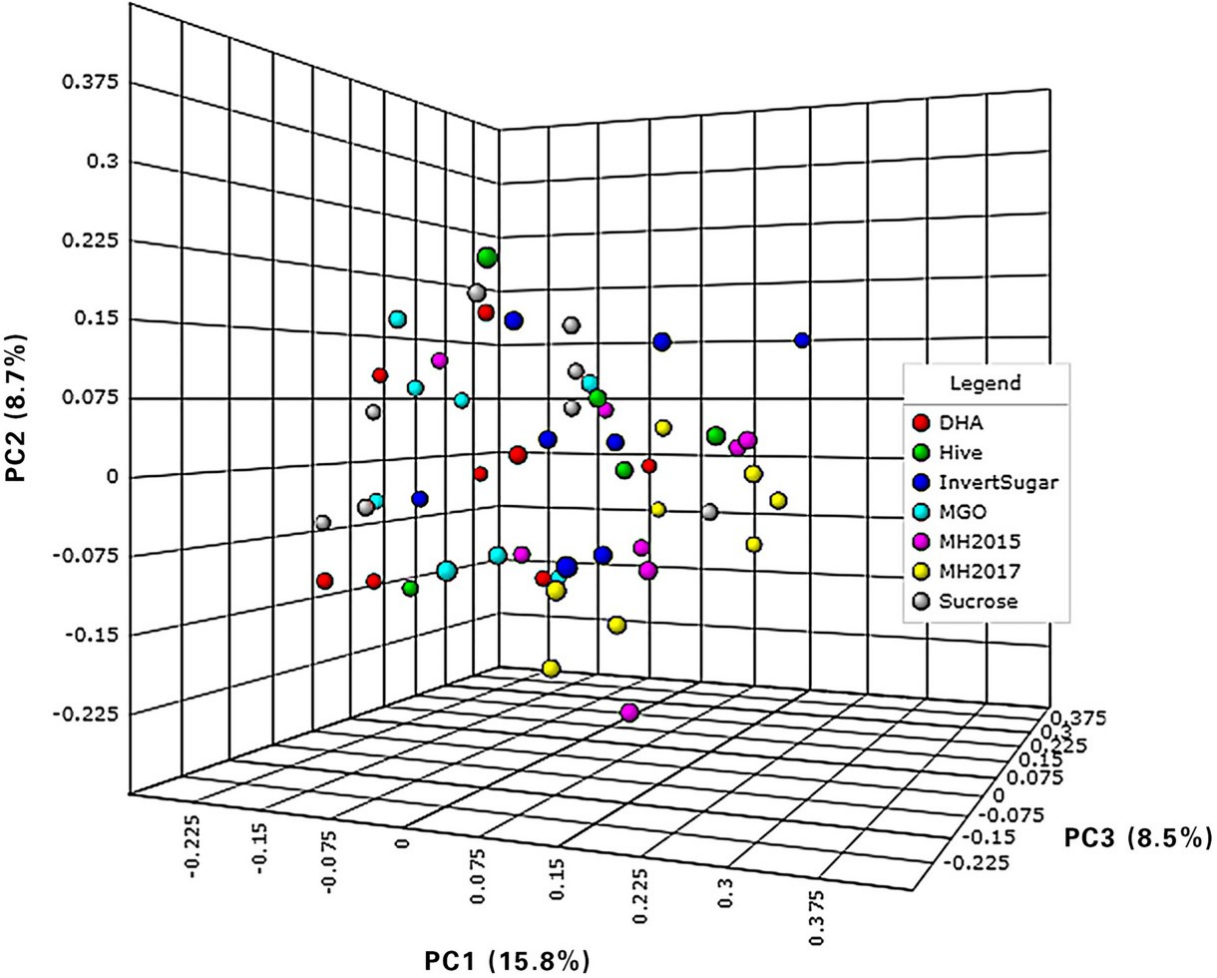
**A principal co-ordinate analysis of the beta-diversity of OTUs within the gut of NZ honey bees.** Honey bees from a single hive were fed one of seven carbohydrate diets for 6 days.

The PCoA visualisation using Bray-Curtis dissimilarity indicated that the majority of the communities showed separation based on the abundance of sucrose (sucrose, MGO, and DHA), or the limitation of sucrose (H, IS, MH15, MH17) (Fig 3) (see S1 Fig for PCoA’s based on different distance methods).

## Discussion

We examined the gut bacteria of adult *A*. *mellifera* from a single colony after being fed seven different dietary regimes for six days. The effect of carbohydrate composition on the diversity and relative abundance of bacteria present in the digestive tract was determined by comparing the effect of invert sugar (mix of monosaccharides) and two different mānuka honeys (predominantly monosaccharides), with the effect of sucrose (a disaccharide), and diets containing the mānuka associated chemicals MGO and DHA in sucrose solutions. These were all compared to the diet that bees consume within a hive.

There was no evidence of correlation between diet and the relative abundance of the five dominant core bacteria in the digestive tract of *A*. *mellifera*. However, the sucrose diet altered the relative abundance of some of the sub-dominant core OTUs when compared with the hive control, and a significant shift in the overall composition of the microbiome was observed.

The relative abundance of Rhizobiaceae increased by 4- to 5-fold, Acetobacteraceae increased by 2- to 3-fold, and *Lactobacillus kunkeei* increased by 2- to 7-fold. In contrast the relative abundance of OTUs from the species *F*. *perrara* decreased with a sucrose diet by 2-fold. *F*. *perrara* is associated with scabbing of the epithelial surface in the pylorus, which is potentially due to an immune response in the bees [65]. All bees were initially exposed to the same hive environment to develop a natural gut microbiome before being fed the specific diet treatment, only the sucrose and DHA treatments appear to have inhibited the proliferation of *F*. *perrara* and potentially the immune system response.

As both sucrose [44, 66], and mānuka honey are antibacterial [49], it was hypothesised that both of these carbohydrate treatments may inhibit the gut bacteria. However, sucrose and mānuka honey appeared to affect the gut bacteria differently as the relative abundances of Rhizobiaceae, Acetobacteraceae, and *L*. *kunkeei* increased with sucrose but this was not observed in the hive controls or the mānuka honey treatments.

These differences in the sub-dominant core bacteria are further evidence that diet affects the bacterial composition within the digestive tracts of *A*. *mellifera*, as already seen with different pollen diets and differing environmental landscapes [22–24]. However, as the dominant core bacteria did not alter, we suggest that the biotic factors affecting the honey bee gut microbiome should be discussed more specifically in terms of dominant or sub-dominant core bacteria, as changes seen so far are relatively subtle and seem to mainly effect the less abundant phylotypes.

The relative abundance of the phylotype *Lactobacillus* sp. (Firm-4) was 3- to 4-fold higher than all other phylotypes, across all treatments. This higher relative abundance did not alter with diet, but since the phylotype *Lactobacillus* sp. contained 25 OTUs that were unable to be classified more finely in our study, it is likely to represent several species. This has previously been shown using 16S rRNA gene sequence analyses, and phenotypic and genetic characteristics to isolate seven species of *Lactobacillus* from the lactic acid bacterial community within bees [67]. Of these seven species, only two were identified in our analysis, *L*. *mellis* and *L*. *mellifer*, suggesting that additional species may feature within our *Lactobacillus* sp. phylotype, and the effect of diet on these individual bacteria may have been concealed, as some may have increased in relative abundance whilst others decreased.

Rhizobiaceae, Acetobacteraceae, and *L*. *kunkeei* are major bacterial phylotypes previously identified in the honey bee crop but absent in the mid- and hindguts of nurse and forager bees [33]. The crop and midgut contain <5% relative abundance of all bacteria in the gut [37], and as expected these bacteria were present in relatively low abundance in our study. This was expected because the digestive tracts of our samples were analysed in their entirety.

In contrast, the dominant core bacteria, which have previously been shown to represent >94% of the gut bacteria in the mid- and hindgut [37], were relatively abundant. Of these, *G. apicola [27]*, *S*. *alvi* [36], *Lactobacillus* sp. [37, 68], and *Bifidobacterium* [68] are likely to efficiently metabolise sugars to extract energy. We observed no large effects of diet on the relative abundances of these dominant core bacteria, despite the variation of sugar type in the diets. Metagenomics analysis, as compared to 16S RNA sequencing, may have identified changes to the bacterial genes in response to the sugar source.

The Acetobacteraceae are a family of primary feeders that break down the di-, oligo- or poly-saccharides such as sucrose to form mono-saccharides that they then metabolise to form acetate and/or lactate [41, 69]. Acetobacteraceae increase in sucrose-rich environments by establishing symbiotic relationships with insects that feed on sugar-rich diets. They have been observed to aid host nutrition [70], increase larval tissue development in the *Anopheles* mosquito [71], and are associated with the defective immune genotype causing *Drosophila* gut disease [72]. Acetobacteraceae Alpha 2.2, recently described as *Parasaccharibacter apium*, is present in the crop of *A*. *mellifera* forager bees, as well as their food stores in the hive, and in the larval gut where they presumably metabolise sucrose to generate acetic acid [33].

Rhizobiaceae are nitrogen-fixing bacteria that may have a pathogenic, symbiotic or saprophytic relationship with the host [73, 74]. Rhizobiaceae, including the species *Ensifer adhaerens* identified in this trial, are predominantly sustained on nitrogen-rich food sources normally because of a paucity of carbohydrates in their environment [73]. *E*. *adhaerens* is a soil bacterium [75] that has not previously been identified in the gut of the honey bee. It is possible that *E*. *adhaerens* was consumed by the bees in this trial if the parent colony had foraged on flowers or water dusted with soil containing this bacterium. The lack of variation in relative abundance of *E*. *adhaerens* between the treatments suggests that either the bacterium was not affected by diet, or were dead within the gut. The fact that the soil bacterium *E*. *adhaerens* was present, supports current literature that bees collect bacteria as they forage [76].

*L*. *kunkeei* are acid-resistant, obligate fructophilic bacteria that produce lactic acid, acetic acid and ethanol [77]. They are the dominant lactic acid bacteria present in honey, bee-collected pollen, and bee bread. They are also present in royal jelly and the honey bee crop [33, 78, 79].

Acetobacteraceae is present in larvae and all nurse worker feeding tissue, suggesting bee larvae acquire bacteria from nurse bees [33]. During larval development, the bacteria undergo ecological succession [35]. For example, the gut of first larval instars of honey bees are dominated by Acetobacteraceae, whereas the fifth instar is dominated by *L*. *kunkeei* [35]. Inoculation with Acetobacteraceae by nurse bees may be an important trigger for this microbial succession. Our study suggests that the relative abundance of Acetobacteraceae is influenced by the sucrose content in the honey bee diet, and so we hypothesise that the worker diet may influence the abundance of Acetobacteraceae in honey bee larvae and this may influence larval and/or adult bee mortality.

During the first three days of larval growth in a colony, the larvae consume a carbohydrate-rich diet containing 18% sugar (sucrose and fructose). The sugar content then increases to 45% for the next two days of larval growth before the cells are capped [80]. Thus, bacteria with saccharolytic activity, especially invertase, dominate the gut of larvae that are exposed to sucrose-rich diets, and this may explain the increase of Acetobacteraceae and *L*. *kunkeei* in the gut of adult bees fed the sucrose-rich diets S, MGO, and DHA. Although some isolates of *P*. *apium* increase larval survival *in vitro* [33], the effect of increasing saccharolytic activity through the feeding of sucrose-rich diets on bee larval development, the microbiome, and ultimately colony health, is unknown. The key metabolites generated by Acetobacteraceae, such as acetate, may have additional physiological effects in the host other than the recently recognised utilisation of organic acids, such as acetate, pyruvate, and succinate, by *S*. *alvi* which reduces oxygen in the ileum to generate a more anaerobic atmosphere [81]. The link between the diet of nurse bees that feed larvae, and the associated effect that this may have on adult bee development was not studied in this trial but should be further researched.

The significant increase of Acetobacteraceae in the gut of adult bees after six days of consuming sucrose-rich diets may be directly related to their ability to break down the disaccharide. As the lifespan of a worker bee averages 15–38 days in summer and >140 days in winter [15, 82], it is likely that *A*. *mellifera* colonies may experience prolonged feeding regimes of sucrose during dearth periods, especially in winter. Prolonged feeding of sucrose may potentially cause a resurgence and transmigration of crop-associated residents further along the digestive tract, potentially resulting in changes to the dominant core bacterial composition within *A*. *mellifera*. While such a bacterial increase may not have any pathogenic implications, an overgrowth of such bacteria may potentially affect the colonisation of the entire microbial community. This overgrowth has been observed in mosquito guts, in which bacterial overgrowth accelerated death [83], and in mice, in which infecting agents and chemical triggers induced intestinal inflammation [84]. The possibility of bacterial overgrowth in honey bees, and any potential implications should be further investigated.

Once the carbohydrates are used, protein substrates obtained from the host, as well as bacterial metabolites and remnant cell debris, enable the growth of nitrogen-fixing bacteria such as Rhizobiaceae [34]. This may explain the increase in Rhizobiaceae observed when the bees in our study were fed sucrose-rich diets. Comparatively, the relative abundance of the dominant core bacteria are likely to remain stable as they are able to utilise other substrates such as nucleosides, flavonoid glycosides, and carboxylic acid [81] that collectively sustain both the host and bacteria.

Monosaccharides and water are rapidly absorbed across the midgut of honey bees. Glucose, the chief energy source for bees, is absorbed within five minutes of consumption, whereas sucrose and fructose must be converted to glucose by host enzymes before absorption can occur [85]. Forager honey bees collect nectar in their crop where invertase (α-glucosidase), the enzyme required for sucrose breakdown, is added [86] from the hypopharyngeal glands (HG) [87, 88]. The HG are most active in nurse bees fed pollen aged 5–15 days as they secrete royal jelly to feed to larvae, which contains protein-rich components and sugar [89, 90]. As the bees in our trial were raised in a colony from 1–10 days it is likely the HG were fully developed [91], and it is therefore possible that they were producing invertase, which may catalyse the breakdown of sucrose in the diet to fructose and glucose.

Several strains of *Bifidobacterium asteroides*, previously identified in the crop of forager bees [68], were not detected in our data. *Bifidobacterium coryneforme*, also previously identified [68], contributed 7–9% of the gut bacteria in all seven diet treatments, although no response to sucrose was observed. *Bifidobacterium* is infrequent in the crop, frequent in the hindgut, and proliferates exclusively on pH neutral media [78]. This sensitivity to acidic conditions may be why *Bifidobacterium* is found in the hindgut [78], rather than the midgut where acid metabolites are generated by sucrose metabolism [92], and thus unaffected by the sucrose treatments.

Although the dominant core bacteria do not require each other to colonise the bee gut, cross-feeding interactions do occur. These interactions may be important for community assembly and its resilience, as illustrated by the large amount of pyruvate produced by *G*. *apicola*, which is utilised by *S*. *alvi* [93]. Similar interactions may also occur among the less abundant members of the community as our results show that the relative abundance of both Acetobactereaceae and *L*. *kunkeei* increase in the presence of a sucrose-rich diet. Although it is unknown whether the increase of these bacteria was in response to each other, an interaction is likely to have occurred because Acetobacteraceae rapidly metabolise sucrose to generate lactate, glucose and fructose, of which the fructose fuels the growth of *L*. *kunkeei*, the latter producing both acetate and lactate [77]. Cross-feeding interactions may also occur between host and bacteria as the major metabolite of Acetobacteraceae is acetate. Acetate serves as an energy source for the growth of the bees, and it is utilised by the dominant core bacteria, such as *S*. *alvi*, to fuel respiratory activity [36]. In rodents, a build-up of acetate, produced by bacteria fed high calorie diets, decreased the pH of the microbial niche, and this in turn caused feedback inhibition of bacteria [94]. At this stage it is unknown whether bacteria in the digestive tract of honey bees fed sucrose-rich diets for extended periods may be associated with this type of feedback loop.

The well-documented *in vitro* antibacterial effects of MGO and DHA (its precursor) were not demonstrated in this trial. As MGO is highly reactive, its half life is short in a biological environment [95] and, therefore, at the time and site of analysis, local concentrations may have been significantly reduced by the time the bees consumed it [95]. Consequently, the MGO may have lost its activity by the time it reached the gastric phase of the digestive tract. MGO may also have denatured in the gut, or perhaps these gut bacteria are simply unaffected by MGO.

Sucrose appears to fuel the rapid proliferation of specific, low-abundance primary feeders such as Rhizobiaceae, as well as Acetobacteraceae and *L*. *kunkeei*. The major metabolites acetate and lactate that are likely to be produced by these bacteria may have important physiological functions, such as weight gain in honey bees [96]. Given the distinct effects of the carbohydrates, a metagenomics-based study would have been useful to examine the alterations in the metabolic functionalities of the microbiome. We did consider functional profiling to infer metabolic capabilities. However, none of the computational approaches currently available [97] were compatible with the customised taxonomic assignation that we used in this study.

In conclusion, we have shown that diet does alter the bacterial composition within the digestive tract of caged adult honey bees. Sucrose-rich diets resulted in the increase of sub-dominant bacteria in the gut of honey bees that produce acetate and lactate metabolites and were associated with significant increases in Acetobacteraceae, Rhizobiaceae, and *L*. *kunkeei*, compared with those fed the sucrose-poor diets. Sucrose-rich diets were also associated with a significant decrease in *F*. *perrara*. Further studies are required to understand the long-term effects of these subtle but significant changes in bacterial composition within the honey bee gut that we observed in response to diet, including the effect of increased metabolites and their effect on larval development, dominant core bacterial composition, and ultimately colony health. The effect of supplementary feeding with sucrose, glucose and other carbohydrates on the metabolism of honey bees will be of great interest to the beekeeping industry which routinely practices supplementary carbohydrate feeding.

## Supporting information

S1 Table Mean total abundance of gut bacteria in NZ honey bees fed different carbohydrate diets for six days. (DOCX)

S2 Table Analysis of Deviance tables and alpha-diversity tables to compare gut bacteria in NZ honey bees fed different carbohydrate diets for six days. (DOCX)

S1 Fig Beta-diversity for gut bacteria in NZ honey bees fed different carbohydrate diets for six days. (DOCX)
