# Supplementary material for: The effect of carbohydrate sources: Sucrose, invert sugar and components of mānuka honey, on core bacteria in the digestive tract of adult honey bees (Apis mellifera)
Source: PLoS One. 2019 Dec 4;14(12):e0225845. doi: 10.1371/journal.pone.0225845 (PMC6892475; doi:10.1371/journal.pone.0225845)
Supplement: S1 Table — (DOCX) [file pone.0225845.s001.docx]

# **S1 Table. Mean total abundance of gut bacteria in NZ honey bees fed different carbohydrate diets.**

| **Treatment** | **Taxa** | **Mean number of unique OTUs** | **Mean total abundance** | **SD** | **Min** | **Max** |
| --- | --- | --- | --- | --- | --- | --- |
| DHA | Acetobacteraceae | 4 | 960 | 727 | 103 | 3100 |
| H | Acetobacteraceae | 4 | 738 | 593 | 158 | 2115 |
| IS | Acetobacteraceae | 3 | 861 | 920 | 26 | 2567 |
| MG | Acetobacteraceae | 4 | 1144 | 853 | 118 | 3122 |
| MH15 | Acetobacteraceae | 4 | 839 | 787 | 107 | 2515 |
| MH17 | Acetobacteraceae | 4 | 740 | 633 | 10 | 2504 |
| S | Acetobacteraceae | 5 | 1390 | 1059 | 118 | 4102 |
| DHA | *Bifidobacterium coryneforme* | 4 | 2262 | 1853 | 346 | 7431 |
| H | *Bifidobacterium coryneforme* | 4 | 2481 | 1981 | 312 | 6686 |
| IS | *Bifidobacterium coryneforme* | 3 | 2322 | 2047 | 88 | 6643 |
| MG | *Bifidobacterium coryneforme* | 4 | 1860 | 1460 | 218 | 5447 |
| MH15 | *Bifidobacterium coryneforme* | 4 | 2559 | 2424 | 272 | 9021 |
| MH17 | *Bifidobacterium coryneforme* | 3 | 2016 | 1420 | 319 | 5012 |
| S | *Bifidobacterium coryneforme* | 4 | 2498 | 2103 | 293 | 8574 |
| DHA | *Ensifer adhaerens* | 1 | 1938 | 624 | 613 | 2684 |
| H | *Ensifer adhaerens* | 1 | 1716 | 1478 | 587 | 4153 |
| IS | *Ensifer adhaerens* | 1 | 1121 | 615 | 386 | 2309 |
| MG | *Ensifer adhaerens* | 1 | 2470 | 1351 | 533 | 4939 |
| MH15 | *Ensifer adhaerens* | 1 | 2091 | 1024 | 447 | 3318 |
| MH17 | *Ensifer adhaerens* | 1 | 1799 | 826 | 284 | 2653 |
| S | *Ensifer adhaerens* | 1 | 2558 | 1045 | 997 | 4232 |
| DHA | *Frischella perrara* | 5 | 1251 | 1729 | 54 | 6186 |
| H | *Frischella perrara* | 5 | 1920 | 2571 | 50 | 10127 |
| IS | *Frischella perrara* | 4 | 1825 | 2601 | 57 | 10185 |
| MG | *Frischella perrara* | 5 | 1363 | 964 | 50 | 3458 |
| MH15 | *Frischella perrara* | 5 | 1741 | 1962 | 7 | 7127 |
| MH17 | *Frischella perrara* | 5 | 1197 | 1356 | 15 | 4024 |
| S | *Frischella perrara* | 5 | 1361 | 2141 | 54 | 9023 |
| DHA | *Gilliamella apicola* | 13 | 1005 | 1521 | 8 | 10546 |
| H | *Gilliamella apicola* | 13 | 1363 | 1815 | 20 | 8639 |
| IS | *Gilliamella apicola* | 13 | 1004 | 1556 | 10 | 11631 |
| MG | *Gilliamella apicola* | 13 | 903 | 1063 | 4 | 5055 |
| MH15 | *Gilliamella apicola* | 13 | 1441 | 2023 | 30 | 11663 |
| MH17 | *Gilliamella apicola* | 13 | 1323 | 1965 | 3 | 10084 |
| S | *Gilliamella apicola* | 13 | 1243 | 1853 | 15 | 13153 |
| DHA | *Lactobacillus* | 25 | 1880 | 2888 | 24 | 13923 |
| H | *Lactobacillus* | 25 | 2063 | 2839 | 9 | 15211 |
| IS | *Lactobacillus* | 25 | 2170 | 3224 | 5 | 22708 |
| MG | *Lactobacillus* | 25 | 2176 | 3248 | 16 | 19835 |
| MH15 | *Lactobacillus* | 25 | 1928 | 2937 | 11 | 21486 |
| MH17 | *Lactobacillus* | 25 | 1893 | 2844 | 29 | 15399 |
| S | *Lactobacillus* | 25 | 2017 | 2979 | 20 | 15304 |
| DHA | *Lactobacillus kunkeei* | 1 | 782 | 741 | 25 | 2078 |
| H | *Lactobacillus kunkeei* | 1 | 519 | 889 | 63 | 2107 |
| IS | *Lactobacillus kunkeei* | 1 | 334 | 586 | 13 | 1650 |
| MG | *Lactobacillus kunkeei* | 1 | 117 | 101 | 32 | 335 |
| MH15 | *Lactobacillus kunkeei* | 1 | 93 | 88 | 11 | 245 |
| MH17 | *Lactobacillus kunkeei* | 1 | 54 | 41 | 23 | 135 |
| S | *Lactobacillus kunkeei* | 1 | 1033 | 1050 | 30 | 2653 |
| DHA | *Lactobacillus mellifer* | 1 | 1625 | 628 | 1122 | 3033 |
| H | *Lactobacillus mellifer* | 1 | 1529 | 397 | 1040 | 2037 |
| IS | *Lactobacillus mellifer* | 1 | 1200 | 548 | 479 | 2005 |
| MG | *Lactobacillus mellifer* | 1 | 1718 | 426 | 1235 | 2276 |
| MH15 | *Lactobacillus mellifer* | 1 | 1690 | 546 | 808 | 2486 |
| MH17 | *Lactobacillus mellifer* | 1 | 1177 | 302 | 826 | 1751 |
| S | *Lactobacillus mellifer* | 1 | 1928 | 339 | 1567 | 2482 |
| DHA | *Lactobacillus mellis* | 8 | 1700 | 1809 | 10 | 6902 |
| H | *Lactobacillus mellis* | 8 | 2005 | 2215 | 25 | 10636 |
| IS | *Lactobacillus mellis* | 7 | 2094 | 2254 | 9 | 8127 |
| MG | *Lactobacillus mellis* | 8 | 1679 | 1892 | 11 | 7789 |
| MH15 | *Lactobacillus mellis* | 6 | 1979 | 2282 | 12 | 9344 |
| MH17 | *Lactobacillus mellis* | 7 | 1727 | 2212 | 13 | 9744 |
| S | *Lactobacillus mellis* | 8 | 2068 | 2126 | 3 | 8015 |
| DHA | Rhizobiaceae | 1 | 6698 | 5289 | 310 | 15273 |
| H | Rhizobiaceae | 1 | 1802 | 2210 | 321 | 5595 |
| IS | Rhizobiaceae | 1 | 1554 | 2931 | 55 | 8716 |
| MG | Rhizobiaceae | 1 | 5230 | 3827 | 587 | 10444 |
| MH15 | Rhizobiaceae | 1 | 2454 | 2982 | 90 | 9242 |
| MH17 | Rhizobiaceae | 1 | 1119 | 1444 | 98 | 4538 |
| S | Rhizobiaceae | 1 | 6296 | 6474 | 1494 | 18307 |
| DHA | *Snodgrassella alvi* | 11 | 770 | 1086 | 1 | 4773 |
| H | *Snodgrassella alvi* | 11 | 1557 | 2345 | 8 | 10789 |
| IS | *Snodgrassella alvi* | 10 | 1160 | 1513 | 4 | 5733 |
| MG | *Snodgrassella alvi* | 11 | 876 | 1118 | 1 | 6361 |
| MH15 | *Snodgrassella alvi* | 11 | 1345 | 2129 | 4 | 10680 |
| MH17 | *Snodgrassella alvi* | 11 | 926 | 1132 | 3 | 5629 |
| S | *Snodgrassella alvi* | 11 | 768 | 1228 | 2 | 5961 |

A treatment is shaded grey if the mean number of OTUs differs from the rest of the treatments.
