# Supplementary material for: The effect of carbohydrate sources: Sucrose, invert sugar and components of mānuka honey, on core bacteria in the digestive tract of adult honey bees (Apis mellifera)
Source: PLoS One. 2019 Dec 4;14(12):e0225845. doi: 10.1371/journal.pone.0225845 (PMC6892475; doi:10.1371/journal.pone.0225845)
Supplement: S2 Table — (DOCX) [file pone.0225845.s002.docx]

# **S2 Table. Analysis of Deviance tables and alpha-diversity tables to compare gut bacteria in NZ honey bees fed different carbohydrate diets for six days.**

# **Table A.** **Analysis of Deviance Table (Poisson)**

Model: poisson, link: log

Response: count

Terms added sequentially (first to last)

| Diversity | Df | Deviance | Resid. Df | Resid. Dev |
| --- | --- | --- | --- | --- |
| Null |  |  | 52 | 0.34768 |
| Treatment | 6 | 0.018121 | 46 | 0.32956 |

Response: count

| Diversity | LR Chisq | Df | Pr(>Chisq) |
| --- | --- | --- | --- |
| Treatment | 0.018121 | 6 | 1 |

# **Table B. Analysis of Deviance Table (Type II Wald chisquare tests)**

Response: log(per.abun)

| Diversity | LR Chisq | Df | Pr(>Chisq) |
| --- | --- | --- | --- |
| Treatment | 24.214 | 6 | 0.0004769*** |
| Phylotype | 2338.043 | 10 | < 2.2e-16 *** |
| Treatment:Phylotype | 187.854 | 60 | 4.265e-15 *** |

Signif. codes: 0 ‘***’ 0.001 ‘**’ 0.01 ‘*’ 0.05 ‘.’ 0.1 ‘ ’ 1

Treatments: DHA, H, IS, MG, MH15, MH17, S

| Max LSD | Min LSD | Ave LSD |
| --- | --- | --- |
| 1.02248 | 0.56003 | 0.60358 |

# **Table C. PERMANOVA**

Permutation test for adonis under NA model, marginal effects of terms

Permutation: free, Number of permutations: 999

adonis2(formula = otus_dist ~ treatment, data = widerep, method = bray, by = "margin")

|  | Df | Sum of Squares | R2 | F | Pr(>F) |
| --- | --- | --- | --- | --- | --- |
| Treatment | 6 | 0.18778 | 0.24327 | 2.4647 | 0.001*** |
| Residual | 46 | 0.58411 | 0.75673 |  |  |
| Total | 52 | 0.77189 | 1.0000 |  |  |

Signif. codes: 0 ‘***’ 0.001 ‘**’ 0.01 ‘*’ 0.05 ‘.’ 0.1 ‘ ’ 1

Table D. Alpha diversity

| Diversity | P - value | Kruskal-Wallis statistic |
| --- | --- | --- |
| Chao1 | 0.0959 | 10.675 |
| Observed OTUs | 0.0959 | 10.675 |
| Shannon | 0.2515 | 7.8125 |
| Simpson | 0.1261 | 9.9661 |
