# Supplementary material for: The effect of carbohydrate sources: Sucrose, invert sugar and components of mānuka honey, on core bacteria in the digestive tract of adult honey bees (Apis mellifera)
Source: PLoS One. 2019 Dec 4;14(12):e0225845. doi: 10.1371/journal.pone.0225845 (PMC6892475; doi:10.1371/journal.pone.0225845)
Supplement: S1 Fig — (DOCX) [file pone.0225845.s003.docx]

S1 Fig. Beta-diversity for gut bacteria in NZ honey bees fed different carbohydrate diets for six days.

Beta diversity

S1 figures A–C display the beta-diversity of OTUs within the gut of NZ honey bees sourced from a single hive that were fed different carbohydrate diets for 6 days. The relative abundance of OTUs were displayed as PCoA plots using different distance matrices.

| 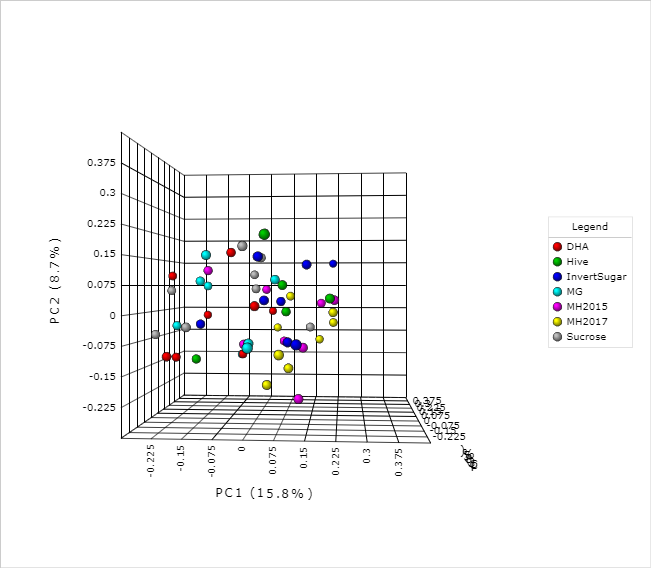 |
| --- |
| Figure A. Bray-Curtis PCoA 3-D visualisation of OTUs. |
| 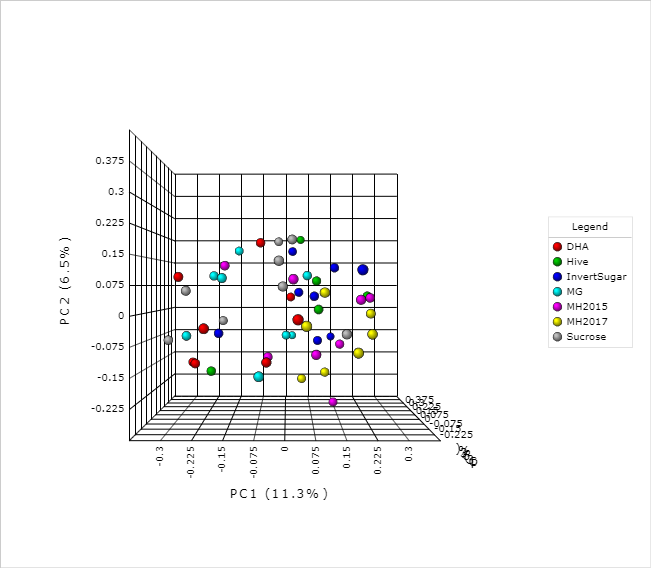 |
| Figure B. Jaccard PCoA 3-D visualisation. |
| 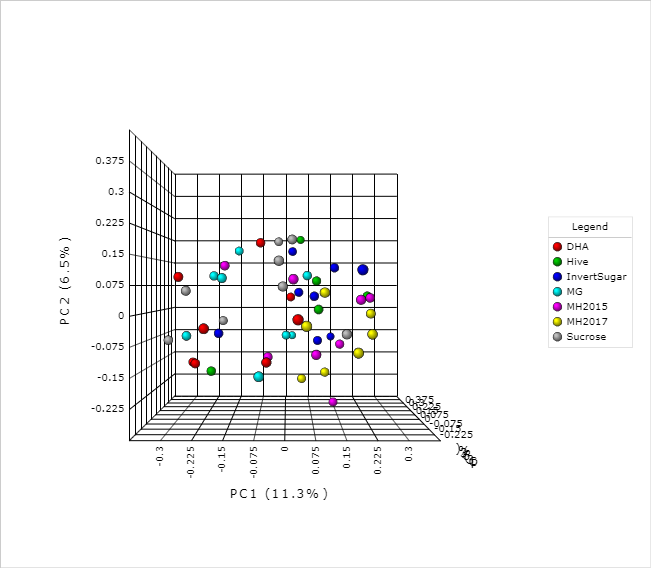 |
| Figure C. Jensen-Shannon PCoA 3-D visualisation. |
